# Evaluating the comparative efficacy of leg cycle ergometry exercise versus conventional physiotherapy on scar healing, muscle strength, functional capacity, and quality of life in coronary artery bypass graft subjects with saphenous vein graft in phase 1: a protocol for randomised controlled trial

**DOI:** 10.1186/s13063-025-09255-1

**Published:** 2025-11-25

**Authors:** Deepali Vinerkar, Vishnu Vardhan

**Affiliations:** Department of Cardiovascular and Respiratory physiotherapy, Ravi Nair physiotherapy college, Datta Meghe Institute of Higher Education and Research, Sawangi (Meghe), 442001, Wardha, India

**Keywords:** CABG, Cardiac rehabilitation, Coronary artery disease, Cycle ergometry, Aerobic exercise

## Abstract

**Background:**

Coronary artery bypass grafting (CABG) is a common surgical treatment for coronary artery disease. The saphenous vein’s advantageous anatomical features make it a popular choice for graft vessels. However, saphenous vein harvesting often results in lower limb complications such as edema, poor scar healing, pain, and reduced functional mobility. These issues can delay recovery and diminish quality of life. Early rehabilitation using leg cycle ergometry may improve outcomes, but comparative evidence with conventional physiotherapy in Phase I post-CABG care is limited.

**Aim:**

To determine the relative benefits of leg cycle ergometry training for CABG patients with saphenous vein graft in phase 1 in terms of scar healing, muscle strength, functional ability, and quality of life.

**Objectives:**

To evaluate the comparative efficacy of leg cycle ergometry exercise versus conventional physiotherapy on scar healing, muscle strength, functional capacity, and quality of life in CABG subjects with saphenous vein grafts during Phase I rehabilitation.

**Methods:**

A randomized controlled trial will be conducted at Shalini Tai Meghe Super Speciality Centre with 70 CABG patients aged 45–65 years. Participants will be randomly allocated to Group A (conventional physiotherapy) and Group B (leg cycle ergometry with physiotherapy) for a 10-day intervention. Outcome measures include the Vancouver Scar Scale, Manual Muscle Testing, 6-Minute Walk Test, and Patient Health Questionnaire-9 (PHQ-9). Data will be analysed using the Mann-Whitney U test and the Wilcoxon signed-rank test; p<0.05 was deemed statistically significant.

**Results:**

As this is a study protocol, no results are available yet. It is hypothesized that leg cycle ergometry, when added to conventional physiotherapy, will lead to greater improvements in scar healing, lower limb strength, functional capacity, and short-term quality of life during Phase I cardiac rehabilitation following CABG surgery.

**Trial registration:**

Clinical Trial Registry-India (CTRI/2025/03/084291). Registered on 15 April 2025.

**Supplementary Information:**

The online version contains supplementary material available at 10.1186/s13063-025-09255-1.

## Introduction

Coronary artery bypass grafting (CABG) is foundational to managing multivessel coronary artery disease. Although saphenous vein grafts (SVGs) may be considered for non-LAD targets, the right ITA (RITA) and radial artery (RA) are associated with improved outcomes [1]. Post-operative cardiopulmonary health is severely compromised with impaired pulmonary and respiratory function mediated by such factors as chest wall pain, decreased respiratory muscle strength, reduced exercise capacity and exercise tolerance, and anxiety with the resumption of more vigorous physical activity [2]. CABG remains one of the most commonly performed cardiac surgeries worldwide, yet post-operative complications continue to influence early recovery and hospital outcomes. Physiotherapy in the ICU was recently incorporated and is starting to work on the concept of early mobilization, as well as its importance in the practice of early mobilization [3]. Structured ICU exercise prescription helps prevent respiratory complications, preserve muscle strength, reduce mortality and disability risk, and promote psychological well-being. The objectives of cardiac rehabilitation (CR) are to promote independence, psychological stability, stress reduction, social reintegration, and restoration of physical capacity [4]. CR is a multidisciplinary, individualized program that addresses the functional and physiological limitations of patients recovering from heart disease [5, 6]. As a fundamental part of every cardiac rehabilitation program (CRP), regular physical activity can improve functional work capacity in cardiac patients after coronary artery bypass graft surgery [7]. Supervised exercise-based cardiac rehabilitation (CR) has been strongly recommended in recent years as an important adjunct therapy following CABG, with well-documented benefits associated with improved prognosis after revascularization [8]. Most usual-care CR in bypass patients is based on aerobic training, and an increasing body of literature has recommended the concomitant application of lower limb resistance training to enhance the beneficial effects of CR, including increased skeletal muscle strength (particularly lower limb muscle volume), an important peripheral factor influencing cardiac function [9]. Despite this, the majority of available research has focused on general aerobic training, with limited attention to targeted lower-limb rehabilitation following saphenous vein graft harvesting. CABG patients who undergo saphenous vein graft harvesting frequently experience lower limb complications such as edema, pain, and delayed scar healing, which can limit mobility and hinder participation in early cardiac rehabilitation. Phase I cardiac rehabilitation refers to the early, in-hospital phase of recovery typically spanning the first 2–10 days for after CABG surgery. It focuses on early mobilization, pulmonary care, and gradual restoration of physical function. While international guidelines such as those from the American Heart Association (AHA) and the European Society of Cardiology (ESC) emphasize early rehabilitation, implementation in developing countries like India remains inconsistent due to limited resources and lack of structured physiotherapy frameworks. Although several international trials have assessed aerobic training after CABG, few have specifically focused on targeted lower-limb rehabilitation addressing donor-site recovery, particularly within early Phase I hospital settings. This evidence gap highlights the need for structured, low-cost interventions such as leg cycle ergometry in resource-limited cardiac units. In the Indian healthcare setting, standardized in-hospital CR protocols are still evolving, highlighting the importance of evaluating feasible, evidence-based interventions during this early recovery. Despite the growing emphasis on early rehabilitation, there is a paucity of randomized controlled trials evaluating structured lower-limb exercises, such as leg cycle ergometry, during Phase I recovery in post-CABG patients.

Application of phase 1 cardiac rehabilitation on patients improved patients’ self-efficacy in terms of independence in daily activities, and it also decreased the need for phase 2 CR [10]. Cycling started earlier in a patient’s ICU stay may further improve patient outcomes [11]. It has been shown that a daily cycling exercise (passive and active) can enhance the functional capacity, self-perceived functional status, and quadriceps muscle strength of ICU patients [12]. Leg cycle ergometry, a low-impact closed-chain exercise, enhances peripheral circulation, promotes venous return, and reduces graft-site complications, thereby facilitating faster scar healing and improving lower-limb strength and endurance during early post-CABG recovery. However, evidence regarding its use during Phase I rehabilitation after CABG with saphenous vein graft is scarce. Therefore, this trial aims to compare leg cycle ergometry with conventional physiotherapy to determine their effects on scar healing, muscle strength, functional capacity, and quality of life in post-CABG patients.

The selected outcome measures—Vancouver Scar Scale (VSS), 6-Minute Walk Test (6MWT), Manual Muscle Testing (MMT), and PHQ-9—were chosen to comprehensively evaluate the physical, functional, and psychological dimensions of recovery. Although other tools like SF-36 or EQ-5D exist, these outcomes are particularly sensitive to short-term Phase I rehabilitation changes. By targeting both early physical recovery and graft-site healing, this study provides a novel and clinically relevant approach to postoperative cardiac rehabilitation, with the potential to inform physiotherapy practices and hospital protocols across similar settings. These outcomes collectively represent the biopsychosocial recovery model essential to modern rehabilitation research.

## Trial design

This study will follow a single-centre, parallel-group, randomized controlled trial design with an allocation ratio of 1:1. Participants will be randomly assigned to either the leg cycle ergometry plus conventional physiotherapy group or the conventional physiotherapy-only group.

The randomization sequence will be computer-generated using block randomization and implemented through sealed, opaque envelopes prepared by an independent researcher who is not involved in participant recruitment or assessment.

Because of the nature of the intervention, participants and treating physiotherapists cannot be blinded. However, all outcome assessors and data analysts will remain blinded to group allocation to reduce measurement bias.

**Fig. 1:**
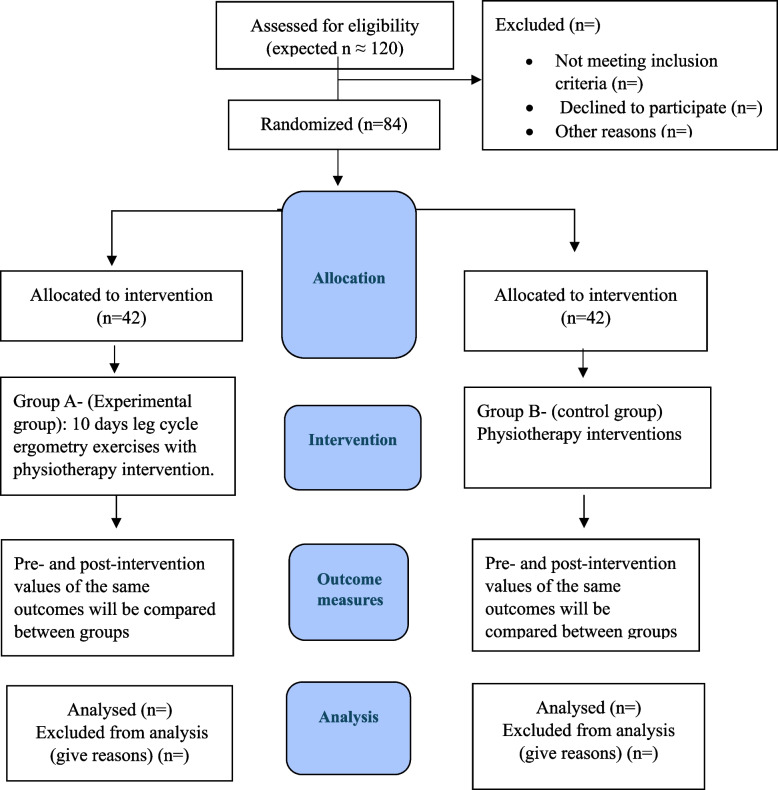
Planned CONSORT flow chart

The study will be conducted at a single tertiary-care teaching hospital with a dedicated cardiac rehabilitation unit. The trial follows the SPIRIT 2013 (Standard Protocol Items: Recommendations for Interventional Trials) guidelines for reporting clinical trial protocols and has been prospectively registered with the Clinical Trial Registry of India (CTRI/2025/03/084291) (Supplementary A). The planned CONSORT flow chart of participant recruitment and randomization is presented in Fig. 1.

Final analysis will include all participants completing the study per protocol and intention-to-treat principles.

This diagram outlines the projected flow of participants through the study in accordance with CONSORT 2010 guidelines. Actual participant numbers and reasons for exclusion will be updated upon study completion.

## Methods

### Study setting

This study protocol follows the SPIRIT 2013 (Standard Protocol Items: Recommendations for Interventional Trials) guidelines for reporting. The trial has been prospectively registered with the Clinical Trial Registry of India (CTRI/2025/03/084291). The study will be conducted after obtaining ethical clearance from the Institutional Ethics Committee at Datta Meghe Institute of Higher Education and Research (DMIHER), Sawangi (Meghe), Wardha, Maharashtra, India. Data collection will take place in the Respiratory Medicine Ward, Shalini Tai Meghe Superspecialist Centre, Sawangi, Wardha, Maharashtra.

#### Inclusion criteria

- Adults aged 45–65 years of either sex.
- Patients who have undergone elective CABG surgery using a saphenous vein graft.
- Hemodynamically stable, conscious, and oriented patients within 1–10 days post-surgery.
- Participants who provide written informed consent.

#### Exclusion criteria

- Hemodynamically unstable patients.
- Patients with neurological, or psychiatric disorders that interfere with rehabilitation participation.
- Patients with post-operative complications requiring re-intubation or prolonged ICU stay (>10 days).

### Sample size calculation

The sample size was calculated in consultation with the institutional biostatistician using Cohen’s formula for comparing two independent means (Cohen, 1988), assuming an effect size (d) = 0.49, α = 0.05, and power (1–β) = 0.80.

Parameter estimates were derived from previous studies with comparable rehabilitation protocols in post-CABG patients [10, 13], the estimated sample size was 35 participants per group. Allowing for a 20% dropout rate, the final sample size was adjusted to 42 participants per group, resulting in a total of 84 participants.

The chosen effect size of 0.49 represents a moderate clinical difference expected in primary outcomes such as functional capacity, scar healing, and quality of life. This sample size is therefore adequate to detect a meaningful difference between groups in early post-CABG recovery.

$$\mathrm{n}=\mathrm{d2}(2(\mathrm{Z}\upalpha /2+\mathrm{Z}\upbeta)2 \cdot (2{\upsigma}2))=35$$

Mean group 1 (M1): 5.53

Standard deviation group 1: 1.75

Mean group 2 (M2): 4.60

Standard deviation group 2: 2.00

Correlation between groups: 0.5 10

Effect size: 0.49

Alpha level: 0.05 Power (1-beta): 0.8

Sample size- 35 in each group

Drop out 20%= 20x35/100=7 35+7=42

The revised sample size is 42 in each group

The total sample size required = 84 subjects (42 subjects in each group)

### Intervention

Interventions will start once patients are hemodynamically stable (typically within 1–2 days post-surgery, usually on postoperative day 2) and will continue for 10 consecutive days during Phase I cardiac rehabilitation. Each session will last 10–15 minutes, conducted twice daily, with exercise progression based on vital stability and patient tolerance. The intervention protocol follows the American Heart Association and ACSM guidelines for early mobilization after cardiac surgery. All sessions will be supervised by certified physiotherapists trained in Phase I cardiac rehabilitation, using standardized checklists to ensure fidelity. Session adherence will be documented daily in participant logbooks. Safety parameters, including heart rate, blood pressure, oxygen saturation (SpO₂), and ECG (when available), will be continuously monitored. Exercise sessions will be immediately terminated in the event of chest pain, SpO₂ <90%, heart rate >120 bpm, arrhythmia, dizziness, or excessive fatigue. Outcome assessors will remain blinded to group allocation to minimize measurement bias.

Any adverse event or serious adverse event (e.g. hypotension, syncope, or wound bleeding) occurring during or after exercise sessions will be documented and reported to the institutional safety committee within 24 hours. Emergency medical support will be available on-site during all sessions.

### Experimental group: (leg cycle ergometry + conventional physiotherapy)

Participants in the experimental group will receive a structured 10-day rehabilitation program combining leg cycle ergometry with conventional physiotherapy. This protocol was modified from Faizan Hamid et al. [10] and Guida et al. (2020).

Initial sessions emphasize breathing control and gentle mobilization to promote circulation and respiratory efficiency. Leg cycle ergometry will begin on day 2 and increase gradually to 15 minutes by day 10, performed twice daily with continuous monitoring. Ambulation, chair transfers, and stepper training are progressively added to enhance lower-limb strength, venous return, and cardiorespiratory endurance. All ergometer sessions will be supervised by the same physiotherapist to ensure uniform progression and adherence.

### Control group

Participants in the control group will follow a 10-day progressive mobilization program based on standard Phase I cardiac rehabilitation [14, 15].

Sessions include diaphragmatic breathing, active limb mobility, chair transfers, stepper training, and ambulation, each performed twice daily for 10–15 minutes per session. Exercise progression will depend on patient tolerance and vital stability (SpO₂ ≥94%, HR ≤110 bpm, Borg RPE ≤13).

Sessions will be conducted under the same supervision and safety monitoring as the experimental group to ensure protocol fidelity.

#### Group A: experimental group (leg cycle ergometry + conventional physiotherapy)[16]

Participants will undergo a 10-day structured exercise program. Each session lasts 10–15 minutes, twice daily, continuously monitoring vital signs. Exercise progression is based on patient tolerance, as detailed in Table 1.

**Table 1:** Day-wise structured protocol for Experimental Group

**Day**	**Exercise protocol**	**Patient position/rationale**
1	• Breathing exercises (1 set of 10 reps) • Active upper and lower limb mobility (1 set of 10 reps)	Supine, head of bed 45° – improve venous return and prevent orthostatic stress
2	• Breathing exercises (1 set of 10 reps) • Active upper and lower limb mobility (1 set of 10 reps) • Standing upright and on-the-spot walking (2 x 1 min) • Leg cycle ergometry (2min)	Supine for ergometer, supported standing for walk – early mobility, circulation
3	• Breathing exercises (1 set of 10 reps) • Active upper and lower limb mobility (1 set of 10 reps) • Standing upright and on-the-spot walking (2 x 1 min) • Ambulation around bed (2-3min) • Leg cycle ergometry(5min)	Edge of bed sitting for chair transfer, supine on ergometer to strengthen lower limbs and functional mobility
4	• Breathing exercises (1 set of 10 reps) • Active upper and lower limb mobility (1 set of 10 reps) • Standing upright and on-the-spot walking (2 x 1 min) • Ambulation around bed (2-3min) • Chair transfer (15min) • Leg cycle ergometry (5min)	Positions same as Day 3. Gradually increasing duration to improve endurance
5	• Breathing exercises (1 set of 10 reps) • Active upper and lower limb mobility (1 set of 10 reps) • Standing upright and on-the-spot walking (2 x 1 min) • Ambulation (15min) • Chair transfer (10-15min) • Leg cycle ergometry (10min)	Focus on progressive functional recovery
6	• Active mobility exercise • Ambulation (10-15min) • Step training on stepper (3–5 min x 2) • Leg cycle ergometry (10 min)	Standing/semi-standing for stepper, supine/seated for ergometer, to improve lower limb strength balance and cardiorespiratory fitness.
7	• Active mobility exercises • Ambulation (10–15 min) • Leg cycle ergometry (10min)	Maintain progressive intensity and endurance.
8	• Active mobility exercises • Stepper training (3–5 min x 2) • Leg cycle ergometry (15 min)	Resistance and ergometer exercise enhance muscle strength and functional capacity
9	• Active mobility exercises • Stepper training (3-5min x 2) • Leg cycle ergometry (15 min)	Resistance and ergometer exercise enhance muscle strength and functional capacity
10	• Active mobility exercise • Stepper training (3-5min x 2) • Leg cycle ergometry (15 min)	Resistance and ergometer exercise enhance muscle strength and functional capacity

#### Group B: control group (conventional physiotherapy)

Participants follow a 10-day progressive mobilization program without leg cycle ergometry. Exercise selection is based on published Phase I cardiac rehabilitation guidelines detailed in Table 2. (Supplementary 2).

**Table 2.** Day-wise structured protocol for Control Group

Day	Exercise Protocol	Patient Position/Rationale
1	• Diaphragmatic breathing (1 set of 10 reps) • Active upper and lower limb mobility (1 set of 10 reps)	Supine, head-of-bed elevated 45° improve circulation, respiratory function
2	• Diaphragmatic breathing (1 set of 10 reps) • Active upper and lower limb mobility (1 set of 10 reps) • Standing upright and on-the-spot walking (2 x 1 min)	Standing with support; early ambulation promotes functional recovery
3	• Diaphragmatic breathing (1 set of 10 reps) • Active upper and lower limb mobility (1 set of 10 reps) • Standing upright and on-the-spot walking (2 x 1 min) • Ambulation around the ward (5 min) • Chair transfer (2 min)	Edge-of-bed sitting, standing for ambulation; improve independence
4	• Diaphragmatic breathing (1 set of 10 reps) • Active upper and lower limb mobility (1 set of 10 reps) • Standing upright and on-the-spot walking (2 x 1 min) • Ambulation around the ward (5 min) • Chair Transfer (5min)	Progressive increase in duration to enhance endurance
5	• Diaphragmatic breathing (1 set of 10 reps) • Active upper and lower limb mobility (1 set of 10 reps) • Standing upright and on-the-spot walking (2 x 1 min) • Ambulation (15 min) • Chair transfer (10 min)	Focus on functional mobility
6	• Active mobility exercises • Step training on the stepper (2 x daily) • Chair transfer (10 min)	Standing/semi-standing; improve lower limb strength and balance
7	• Active mobility exercise • Step training (2 x daily) • Chair transfer (15 min)	Maintain intensity and improve confidence in movement
8	• Active mobility exercises • Stepper training (2 x daily) • Resistance exercises (2 x 10 min)	Strengthening the lower limb and functional independence
9	• Active mobility exercise • Stepper training (2 x daily) • Resistance exercises (2 x 10 min)	Progressive overload for strength
10	• Active mobility exercises • Stepper training (2 x daily) • Resistance exercise (2 x 10 min)	Progressive overload for functional gains

Exercises will be paused or discontinued if any adverse symptoms appear, and patients will be re-evaluated before resuming activity.

#### Equipment required

The leg ergometer used in this trial is designed for supine and seated exercise, allowing safe application in early post-operative cardiac patients where standard cycle ergometry is unsuitable.

Its compact design, adjustable resistance, and ease of bedside positioning make it particularly useful for intensive care and Phase I cardiac rehabilitation settings [17].

#### Instrument description

Device: Portable Pedal Ergometer

Model: MediCycle-LCE

Manufacturer: Mediverse Healthcare Pvt Ltd, India

Type: Manual leg cycle ergometer, usable in supine or sitting positions

Features: Non-slip pedals with adjustable foot straps, digital display (time, RPM, distance), manual resistance knob (0–5 levels)

Reliability & Reproducibility: Demonstrated ICC >0.85 for repeated use in ICU/early cardiac rehabilitation settings [10, 13]

Administrability: Compact, safe for bedside use, suitable for both semi-recumbent and seated patients

All ergometer sessions will be supervised by trained physiotherapists, ensuring standardized resistance progression and patient safety during use.



#### Outcome measures

All outcomes will be assessed at two time points: baseline (post-operative day 1–2, before intervention) and at the end of the 10-day Phase I rehabilitation period (post-operative day 10). Assessments will be conducted by blinded physiotherapists in the hospital setting.

This trial has two co-primary outcomes: (1) scar healing measured by the Vancouver Scar Scale and (2) functional capacity measured by the 6-Minute Walk Test. Secondary outcomes include muscle strength (MMT) and quality of life (PHQ-9).

#### Primary outcome measures

1. Scar Healing (Vancouver Scar Scale): The Vancouver Scar Scale assesses four scar parameters—height, pliability, pigmentation, and vascularity—each scored to yield a total of 0–13, where higher scores indicate greater impairment. It is one of the earliest validated clinical tools for objective evaluation of post-surgical scars and is sensitive to short-term healing responses in hospital settings.
2. Functional Capacity (6-Minute Walk Test): The 6MWT is a standardized sub-maximal exercise test that evaluates aerobic capacity and endurance. The total distance walked in six minutes reflects overall cardiopulmonary fitness. It is widely recommended in cardiac rehabilitation guidelines (AHA/ESC) for assessing early functional gains in post-CABG patients

#### Secondary outcome measures

1. Quality of life-PHQ-9: The Patient Health Questionnaire-9 (PHQ-9) is a validated screening tool for depressive symptoms. Scores of 5–9, 10–14, 15–19, and ≥20 indicate mild, moderate, moderately severe, and severe depression, respectively. This measure captures the psychological dimension of recovery, which can influence engagement and adherence during early rehabilitation.
2. Manual muscle testing: Manual Muscle Testing assesses voluntary muscle strength against graded resistance (Grades 0–5). The examiner stabilizes the limb and applies resistance to determine maximal effort. It provides a practical, reproducible assessment of lower-limb strength when instrument-based testing is not feasible in post-surgical patients.

Together, these outcome measures represent the biopsychosocial recovery model, capturing scar healing (tissue integrity), functional performance (6MWT), muscular strength (MMT), and emotional well-being (PHQ-9). The chosen tools are particularly suitable for short-term, hospital-based Phase I cardiac rehabilitation and have established validity and reliability in this population.

## Discussion

Coronary artery bypass grafting remains one of the most performed and effective revascularization procedures, yet donor-site complications from saphenous vein harvesting such as edema, pain and delayed scar healing can significantly restrict early mobility and delay recovery. Early rehabilitation and mobilization are vital to reduce postoperative functional decline and improve hospital outcomes. Leg cycle ergometry, a closed chain rhythmic exercise, can enhance venous return, improve peripheral circulation, and maintain muscle activation without imposing excess cardiac stress. Incorporating LCE during Phase I cardiac rehabilitation may therefore promote graft site healing, preserve lower limb strength and accelerate functional independence. The selected outcome measures—Vancouver Scar Scale (VSS), 6-Minute Walk Test (6MWT), Manual Muscle Testing (MMT), and PHQ-9—collectively capture the multidimensional nature of recovery. VSS evaluates donor site tissue integrity, 6MWT and MMT reflect physiological and functional capacity, while PHQ-9 captures short-term psychological well-being that influences adherence to rehabilitation. Earlier studies, such as those by Hirschhorn et al. [13] and Hamid et al. [10], demonstrated the safety and feasibility of early ergometry in CABG patients but did not assess localized donor-site outcomes or psychological domains. Hence, this trial addresses an important evidence gap by integrating lower-limb-specific intervention and comprehensive outcome evaluation within the early post-CABG recovery phase, especially in an Indian tertiary-care setting where structured Phase I cardiac rehabilitation is still developing.

This study’s strengths lie in its randomized, assessor-blinded design, use of standardized intervention checklists, and continuous safety monitoring to ensure patient protection and methodological rigor. However, limitations such as the single-center setting, small sample size, and 10-day intervention period may restrict generalizability and long-term interpretation. The inability to blind participants and therapists is inherent to exercise trials, but potential bias will be minimized through blinded assessors, uniform exercise protocols, and inter-rater reliability training. Despite these limitations, the study provides a feasible, cost-effective rehabilitation model that can be readily implemented in resource-limited cardiac units. If the findings confirm the hypothesized benefits, leg cycle ergometry could be incorporated as a routine component of early Phase I cardiac rehabilitation to enhance physical recovery, graft-site healing, and psychological outcomes. Future multicentre trials with extended follow-up are warranted to validate durability of effects and to establish broader clinical applicability. By establishing early-phase evidence from an Indian tertiary-care center, this trial contributes to the growing global focus on context-specific, low-cost cardiac rehabilitation models that address both physiological and psychosocial recovery. Future multicenter trials with extended follow-up are warranted to validate the durability and generalizability of these findings.

### Recruitment

Subjects will be recruited through the Cardiovascular and Thoracic Surgery (CVTS) Intensive Care Unit and Ward, Shalini Tai Meghe Super speciality Centre, Sawangi (Meghe), Wardha, Maharashtra, India. Enrollment into the trial and baseline assessments will occur prior to the initiation of intervention. Eligible patients meeting the inclusion criteria will be approached within 24–48 hours of achieving hemodynamic stability following coronary artery bypass grafting. The purpose, procedures, potential benefits, and risks of the study will be clearly explained, and written informed consent will be obtained from all participants prior to recruitment.

**Fig. 2:**
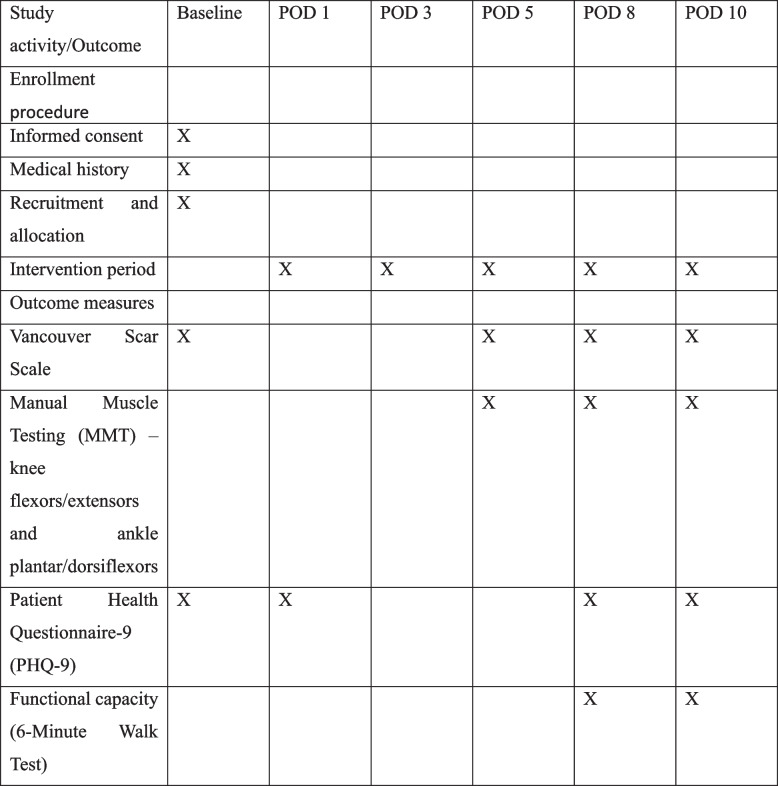
SPIRIT Schedule of enrollment, intervention, and outcome measures

Recruitment will continue until the required sample size of 84 participants (42 per group) has been achieved. The schedule of enrollment, intervention, and assessments is presented in Fig. 2.

### Randomization

#### Sequence generation

Participants will be randomized in a 1:1 ratio to either the experimental or control group using computer-generated block randomization created by an independent statistician not involved in patient enrollment or assessment.

#### Allocation Concealment Mechanisms

Allocation will be concealed using the sequentially numbered, opaque, sealed envelopes (SNOSE) method. Physiotherapist will label and store the envelopes securely. Upon subject enrollment, the next envelope in sequence will be opened to reveal group allocation. This ensures allocation concealment and prevents selection bias.

#### Implementation

The allocation sequence will be generated by the biostatistician, while participant enrollment will be performed by the primary investigator. Group assignment will be carried out by the therapist after eligibility confirmation. The physiotherapists responsible for delivering interventions will not have access to the randomization list.

#### Blinding

Due to the nature of the intervention, participant and therapist blinding is not feasible because subjects will know whether they are performing leg cycle ergometry. However, outcome assessors (evaluating scar healing, muscle strength, functional capacity, and psychological outcomes) and data analysts will remain blinded to group allocation to minimize measurement and analysis bias.

To ensure assessor reliability, standardized training and checklists will be used for all outcome measures.

### Data collection method

All participants will be enrolled and baseline data collected on post-operative day 1–2, followed by a 10-day in-hospital Phase I cardiac rehabilitation program (post-operative day 2–11). Assessments will be performed by blinded physiotherapists at the specified time points according to the SPIRIT 2013 schedule shown in Fig. 2 (Supplementary no.3).

### Data management

Data collection and reporting will be overseen by the principal investigator and verified by an independent quality monitor. All research documentation will be cross-checked twice for accuracy. After data collection, groups will remain blinded during data entry and cleaning. The final Excel dataset will be reviewed by an allocation-blinded statistician for analysis. Data will be stored in a secure, password-protected, access-controlled server located at Datta Meghe Institute of Higher Education and Research.

Participant data will be anonymized using coded identifiers, and no personal details will appear in reports or publications. Backup copies will be maintained on encrypted drives to prevent data loss.

Participation is voluntary; participants may withdraw at any time. If consent to use existing data is denied, such data will be permanently deleted.

### Statistical methods

All statistical analyses will be performed using IBM SPSS Statistics version or equivalent software. Descriptive statistics will be used to summarize demographic and baseline characteristics, with continuous variables expressed as mean ± standard deviation and categorical variables presented as frequencies and percentages. The Shapiro–Wilk test will be applied to assess data normality. For within-group comparisons (pre- and post-intervention), the paired t-test will be used for normally distributed data, while the Wilcoxon signed-rank test will be applied for non-parametric data. Between-group comparisons (experimental versus control) will be conducted using the independent t-test for normally distributed data and the Mann–Whitney U test for non-parametric data. Analysis of covariance (ANCOVA) will be employed to adjust for any baseline differences between groups. Missing data will be addressed using an intention-to-treat approach, applying the last observation carried forward method where appropriate. In addition, subgroup analyses may be performed based on age, sex, and baseline functional capacity to explore potential secondary interactions. A p-value of <0.05 will be considered statistically significant for all analyses.

### Data monitoring

The Data Monitoring Committee will oversee recruitment progress, protocol adherence, and safety reporting throughout the trial.

### Trial status

- Recruitment began Date- June 2025
- Expected recruitment completion Date- August 2026
- Protocol Version: 1.0
- Amendment History: None

## Supplementary Information

Supplementary Material 1.

Supplementary Material 2.

Supplementary Material 3.

Supplementary Material 4.
