# Supplementary material for: Evaluating the comparative efficacy of leg cycle ergometry exercise versus conventional physiotherapy on scar healing, muscle strength, functional capacity, and quality of life in coronary artery bypass graft subjects with saphenous vein graft in phase 1: a protocol for randomised controlled trial
Source: Trials. 2025 Nov 25;26:545. doi: 10.1186/s13063-025-09255-1 (PMC12649097; doi:10.1186/s13063-025-09255-1)
Supplement: Supplementary file 1 — Supplementary Material 1. [file 13063_2025_9255_MOESM1_ESM.docx]

**Supplement no.1 CONSORT flow chart**

Assessed for eligibility (expected n ≈ 120)

Excluded (n=)

 Not meeting inclusion criteria (n=)

Declined to participate (n=)

Other reasons (n=)

 Declined to participate (n=)

 Other reasons (n=)

## **Allocation**

Randomized (n=84)

Allocated to intervention (n=42)

Allocated to intervention (n=42)

Group B- (control group) Physiotherapy interventions

Group A- (Experimental group): 10 days leg cycle ergometry exercises with physiotherapy intervention.

## **Intervention**

## **Outcome measures**

Pre- and post-intervention values of the same outcomes will be compared between groups

Pre- and post-intervention values of the same outcomes will be compared between groups

## **Analysis**

Analysed (n=)
 Excluded from analysis (give reasons) (n=)

Analysed (n=)
Excluded from analysis (give reasons) (n=)

**Figure 1. Planned CONSORT flow chart**
