# Supplementary material for: Evaluating the comparative efficacy of leg cycle ergometry exercise versus conventional physiotherapy on scar healing, muscle strength, functional capacity, and quality of life in coronary artery bypass graft subjects with saphenous vein graft in phase 1: a protocol for randomised controlled trial
Source: Trials. 2025 Nov 25;26:545. doi: 10.1186/s13063-025-09255-1 (PMC12649097; doi:10.1186/s13063-025-09255-1)
Supplement: Supplementary file 2 — Supplementary Material 2. [file 13063_2025_9255_MOESM2_ESM.docx]

**Supplementary no.2 Exercise Protocol**

| **Day** | **Exercise Protocol** | **Patient position / Rationale** |
| --- | --- | --- |
|  |  |  |
| 1 | - Breathing exercises (1 set of 10 reps) - Active upper and lower limb mobility (1 set of 10 reps) | Supine, head of bed 45° – improve venous return and prevent orthostatic stress |
| 2 | - Breathing exercises (1 set of 10 reps) - Active upper and lower limb mobility (1 set of 10 reps) - Standing upright and on-the-spot walking (2 x 1 min) - Leg cycle ergometry (2min) | Supine for ergometer, supported standing for walk – early mobility, circulation |
| 3 | - Breathing exercises (1 set of 10 reps) - Active upper and lower limb mobility (1 set of 10 reps) - Standing upright and on-the-spot walking (2 x 1 min) - Ambulation around bed (2-3min) - Leg cycle ergometry(5min) | Edge of bed sitting for chair transfer, supine on ergometer to strengthen lower limbs and functional mobility |
| 4 | - Breathing exercises (1 set of 10 reps) - Active upper and lower limb mobility (1 set of 10 reps) - Standing upright and on-the-spot walking (2 x 1 min) - Ambulation around bed (2-3min) - Chair transfer (15min) - Leg cycle ergometry (5min) | Positions same as Day 3. Gradually increasing duration to improve endurance |
| 5 | - Breathing exercises (1 set of 10 reps) - Active upper and lower limb mobility (1 set of 10 reps) - Standing upright and on-the-spot walking (2 x 1 min) - Ambulation (15min) - Chair transfer (10-15min) - Leg cycle ergometry (10min) | Focus on progressive functional recovery |
| 6 | - Active mobility exercise - Ambulation (10-15min) - Step training on stepper (3-5 min x 2) - Leg cycle ergometry (10 min) | Standing /semi-standing for stepper, supine /seated for ergometer, to improve lower limb strength balance and cardiorespiratory fitness. |
| 7 | - Active mobility exercises - Ambulation (10-15 min) - Leg cycle ergometry (10min) | Maintain progressive intensity and endurance. |
| 8 | - Active mobility exercises - Stepper training (3-5 min x 2) - Leg cycle ergometry (15 min) | Resistance and ergometer exercise enhance muscle strength and functional capacity |
| 9 | - Active mobility exercises - Stepper training (3-5min x 2) - Leg cycle ergometry (15 min) | Resistance and ergometer exercise enhance muscle strength and functional capacity |
| 10 | - Active mobility exercise - Stepper training (3-5min x 2) - Leg cycle ergometry (15 min) | Resistance and ergometer exercise enhance muscle strength and functional capacity |

**Table1. Day-wise structured protocol for Experimental Group**

**Group B: Control group (Conventional Physiotherapy)**

| Day | Exercise Protocol | Patient Position/ Rationale |
| --- | --- | --- |
| 1 | - Diaphragmatic breathing (1 set of 10 reps) - Active upper and lower limb mobility (1 set of 10 reps) | Supine, head-of-bed elevated 45^o^ improve circulation, respiratory function |
| 2 | - Diaphragmatic breathing (1 set of 10 reps) - Active upper and lower limb mobility (1 set of 10 reps) - Standing upright and on-the-spot walking (2 x 1 min) | Standing with support; early ambulation promotes functional recovery |
| 3 | - Diaphragmatic breathing (1 set of 10 reps) - Active upper and lower limb mobility (1 set of 10 reps) - Standing upright and on-the-spot walking (2 x 1 min) - Ambulation around the ward (5 min) - Chair transfer (2 min) | Edge-of-bed sitting, standing for ambulation; improve independence |
| 4 | - Diaphragmatic breathing (1 set of 10 reps) - Active upper and lower limb mobility (1 set of 10 reps) - Standing upright and on-the-spot walking (2 x 1 min) - Ambulation around the ward (5 min) - Chair Transfer (5min) | Progressive increase in duration to enhance endurance |
| 5 | - Diaphragmatic breathing (1 set of 10 reps) - Active upper and lower limb mobility (1 set of 10 reps) - Standing upright and on-the-spot walking (2 x 1 min) - Ambulation (15 min) - Chair transfer (10 min) | Focus on functional mobility |
| 6 | - Active mobility exercises - Step training on the stepper (2 x daily) - Chair transfer (10 min) | Standing/semi-standing; improve lower limb strength and balance |
| 7 | - Active mobility exercise - Step training (2 x daily) - Chair transfer (15 min) | Maintain intensity and improve confidence in movement |
| 8 | - Active mobility exercises - Stepper training (2 x daily) - Resistance exercises (2 x 10 min) | Strengthening the lower limb and functional independence |
| 9 | - Active mobility exercise - Stepper training (2 x daily) - Resistance exercises (2 x 10 min) | Progressive overload for strength |
| 10 | - Active mobility exercises - Stepper training (2 x daily) - Resistance exercise (2 x 10 min) | Progressive overload for functional gains |

**Table 2. Day-wise structured protocol for Control Group**

(Supplementary no.2)
