# Supplementary material for: Evaluating the comparative efficacy of leg cycle ergometry exercise versus conventional physiotherapy on scar healing, muscle strength, functional capacity, and quality of life in coronary artery bypass graft subjects with saphenous vein graft in phase 1: a protocol for randomised controlled trial
Source: Trials. 2025 Nov 25;26:545. doi: 10.1186/s13063-025-09255-1 (PMC12649097; doi:10.1186/s13063-025-09255-1)
Supplement: Supplementary file 3 — Supplementary Material 3. [file 13063_2025_9255_MOESM3_ESM.docx]

**Supplementary no.3 Data collection method:**

| Study activity/Outcome | Baseline | POD 1 | POD 3 | POD 5 | POD 8 | POD 10 |
| --- | --- | --- | --- | --- | --- | --- |
| Enrollment  procedure |  |  |  |  |  |  |
| Informed consent | X |  |  |  |  |  |
| Medical history | X |  |  |  |  |  |
| Recruitment and allocation | X |  |  |  |  |  |
| Intervention period |  | X | X | X | X | X |
| Outcome measures |  |  |  |  |  |  |
| Vancouver Scar Scale | X |  |  | X | X | X |
| Manual Muscle Testing (MMT) – knee flexors/extensors and ankle plantar/dorsiflexors |  |  |  | X | X | X |
| Patient Health Questionnaire-9 (PHQ-9) | X | X |  |  | X | X |
| Functional capacity (6-Minute Walk Test) |  |  |  |  | X | X |

Figure 2. SPIRIT Schedule of enrollment, intervention, and outcome measures.

(Supplementary no.3)
