# Supplementary material for: Evaluating the comparative efficacy of leg cycle ergometry exercise versus conventional physiotherapy on scar healing, muscle strength, functional capacity, and quality of life in coronary artery bypass graft subjects with saphenous vein graft in phase 1: a protocol for randomised controlled trial
Source: Trials. 2025 Nov 25;26:545. doi: 10.1186/s13063-025-09255-1 (PMC12649097; doi:10.1186/s13063-025-09255-1)
Supplement: Supplementary file 4 — Supplementary Material 4. [file 13063_2025_9255_MOESM4_ESM.docx]

**CONSENT FORM**

CONSENT FORM EVALUATING THE COMPARATIVE EFFICACY OF LEG CYCLE ERGOMETR EXERCISE VERSUS CONVENTIONAL PHYSIOTHERAPY INTERVENTION ON SCAR HEALING, MUSCLE STRENGTH, FUNCTIONAL CAPACITY AND QUALITY OF LIFE IN CORONARY ARTERY BYPASS GRAFTING SUBJECTS WITH SAPHENOUS VEIN GRAFT IN PHASE I: A RANDOMIZED CONTROLLED TRIAL

This is to certify that I…………………………………………………………………. have been given that required information with respect to my participation as a volunteer in the above-mentioned study. The contents of form have been explained to me in my own language.

I confirm that I will receive a signed copy of consent form. I have understood the nature of the study and I allow my (relationship) Name: to be part of this research study as a subject.

Name…………………… Age/Gender: …………………

Address: ………………………… Contact No.: ………………….

Date: / / Place: …………………………

Sign: …………………………

I undersigned Dr. Deepali Vinerkar (PT), have explained the study details and have cleared all the queries put forth by the above volunteers to the best of my ability. I confirm that all data and test results achieved will be kept strictly confidential and will be withheld from any misuse.

Date: / /
